# Acetaldehyde reinforcement and motor reactivity in newborns with or without a prenatal history of alcohol exposure

**DOI:** 10.3389/fnbeh.2013.00069

**Published:** 2013-06-17

**Authors:** Samanta M. March, Marcela E. Culleré, Paula Abate, José I. Hernández, Norman E. Spear, Juan C. Molina

**Affiliations:** ^1^Laboratorio de Alcohol, Ontogenia y Desarrollo, Instituto de Investigación Médica Mercedes y Martín FerreyraCórdoba, Argentina; ^2^Facultad de Psicología, Universidad Nacional de Córdoba, Cátedra Psicobiología ExperimentalCórdoba, Argentina; ^3^Center for Developmental Psychobiology, Department of Psychiatry, Binghamton UniversityBinghamton, NY, USA

**Keywords:** prenatal ethanol exposure, acetaldehyde, associative learning, reinforcement, ontogeny, neonatal learning, motor activity, tolerance

## Abstract

Animal models have shown that early ontogeny seems to be a period of enhanced affinity to ethanol. Interestingly, the catalase system that transforms ethanol (EtOH) into acetaldehyde (ACD) in the brain, is more active in the perinatal rat compared to adults. ACD has been found to share EtOH's behavioral effects. The general purpose of the present study was to assess ACD motivational and motor effects in newborn rats as a function of prenatal exposure to EtOH. Experiment 1 evaluated if ACD (0.35 μmol) or EtOH (0.02 μmol) supported appetitive conditioning in newborn pups prenatally exposed to EtOH. Experiment 2 tested if prenatal alcohol exposure modulated neonatal susceptibility to ACD's motor effects (ACD dose: 0, 0.35 and 0.52 μmol). Experiment 1 showed that EtOH and ACD supported appetitive conditioning independently of prenatal treatments. In Experiment 2, latency to display motor activity was altered only in neonates prenatally treated with water and challenged with the highest ACD dose. Prenatal EtOH experience results in tolerance to ACD's motor activity effects. These results show early susceptibility to ACD's appetitive effects and attenuation of motor effects as a function of prenatal history with EtOH, within a stage in development where brain ACD production seems higher than later in life.

## Introduction

Epidemiological studies have shown an association between prenatal exposure to ethanol (EtOH) and later development of EtOH drinking problems (Baer et al., 2003; Alati et al., 2006). Animal studies have extensively agreed with this observation (for a review see Abate et al., 2008). The effect of prenatal exposure to EtOH upon later responsiveness to the drug has been observed soon after birth (March et al., 2009; Miranda-Morales et al., 2010), during infancy (Arias and Chotro, 2005), adolescence (Chotro and Arias, 2003), and adulthood (Barbier et al., 2008).

Several mechanisms have been proposed to explain this effect. One possibility relies on fetal associative learning and memory capabilities. The odor, taste and somatosensory stimulation provided by EtOH in the amniotic fluid is perceived by the fetus (Domínguez et al., 1996). The fetus can also experience EtOH's postabsorptive pharmacological effects (Domínguez et al., 1996; Abate et al., 2000), originating the opportunity for the development of an association between EtOH's chemosensory properties and its pharmacological effects (Abate et al., 2000, 2001, 2002). Additionally, it has been proposed that exposure to EtOH during early ontogeny involves learning about positive effects of the drug, since at this developmental stage rats seem more susceptible to EtOH's effects than later in life (Molina et al., 2007b; Pautassi et al., 2009). In other words, there seems to be an ontogenetic switch in the way organisms perceive EtOH's positive effects. For example, EtOH intoxication on postnatal days 7–8 increases EtOH intake and enhances EtOH's palatability. On the contrary, intoxication during postnatal days 10–11 decreases EtOH intake and increases aversive responses to the drug (Arias and Chotro, 2006).

These changes in EtOH consumption and reinforcement occur during a period of rapid brain maturation (Pautassi et al., 2009). Among the several changes occurring in the neural system during early ontogeny are those concerning EtOH metabolism. Although some sources of EtOH metabolism are slow to develop, brain catalase activity and acetaldehyde (ACD) production are significantly higher in rat pups than adults (Gill et al., 1992). Catalase activity progressively drops during the first weeks of life (Del Maestro and McDonald, 1987). This is not a minor fact, since central catalase oxidizes EtOH into ACD in the brain (Aragon et al., 1991). Additionally, in adult rodents ACD has been shown to have an active role in EtOH postabsorptive effects, such as motor stimulation, anxiolysis, appetitive, and aversive properties (Correa et al., 2003a,b, 2009; Escarabajal et al., 2003; Quertemont et al., 2003). In spite of this evidence, few studies have analyzed ACD effects during infancy (Nizhnikov et al., 2007; Pautassi et al., 2011). These studies show that appetitive memories related to EtOH can be blocked by sequestering ACD (Pautassi et al., 2011; March et al., 2013) or by the inhibition of the catalase system (Nizhnikov et al., 2007).

An important issue in studying the effects of EtOH (or ACD) during early ontogeny is the accommodation of research tools to the age-specific behavioral repertoire of the animal. Suckling is an age-specific, complex and organized behavior, essential for the newborn's survival (Petrov et al., 2003). The artificial nipple technique has provided valuable information about the reinforcing properties of fluids during early ontogeny (Nizhnikov et al., 2002). Neonates readily self-administer milk as well as sucrose and EtOH solutions through the surrogate nipple. Exposure to an artificial nipple providing EtOH increases subsequent attachment to an empty surrogate nipple (Cheslock et al., 2001). Additionally, 2–5-h old pups are also capable of robust olfactory conditioning when an odor cue (conditioned stimulus, CS) is associated with a natural reinforcer such as milk (Cheslock et al., 2000). For tests of EtOH reinforcement, presenting a surrogate nipple providing water (CS) in close temporal contiguity with an i.p. administration of very low EtOH doses (0.125 and 0.25 g/kg) results in increased attachment to an empty surrogate nipple (Petrov et al., 2003). Although prenatal exposure to EtOH increases later acceptance for the drug (Arias and Chotro, 2005; March et al., 2009), it has not been established if this experience alters the acute effects induced by EtOH's first metabolite (ACD).

EtOH motivational and motor effects have been linked (Arias et al., 2010). Moreover, motor activating effects of EtOH seems to be mediated by ACD, in adult and infant rodents (Correa et al., 2003a,b; Pautassi et al., 2011). Additionally, infants are likely to exhibit motor conditioned responses resulting from the association between chemosensory stimuli and drugs of abuse, such as EtOH that alter frequency and duration of different behaviors. These learned responses may overshadow other specific behaviors (such as suckling) indicative of the drug's motivational properties.

The general purpose of the present study was to analyze ACD's reinforcement capability as well as its motor effects in newborn pups, as a function of prenatal exposure to EtOH. In Experiment 1, this goal was addressed using the artificial nipple technique, an animal model developed to assess drug and natural reinforcement in neonatal pups (Petrov et al., 2003). Yet, the use of EtOH (or ACD) as an unconditioned stimulus (US), can also lead to motor conditioned responses which can confound interpretations in tests related with drug reinforcement (Molina et al., 2007a). For this reason, Experiment 2 was performed to directly assess the possibility that ACD may induce motor effects that can alter pups' responsiveness to the artificial nipple.

## Methods

### Subjects

Wistar derived rats were born and reared at the vivarium of the Instituto de Investigaciones Médicas Mercedes y Martín Ferreyra. Temperature was kept at 22–24°C with a 12-h light/12-h dark cycle (light onset at 0800 h). Vaginal smears of female rats (pregnancy weight: 230–300 g) were microscopically analyzed on a daily basis. On the day of Proestrus, females were housed with males (3 females per male) overnight. The next morning, females were withdrawn and this day was considered to be gestational day 0 (GD 0). From DG 0, pregnant females were housed in group of 3 in standard maternity cages. These dams had continuous access to rat chow (Cargill, Buenos Aires, Argentina) and tap water. From GD 17 to 21, dams were individually housed.

Animals were maintained and treated in compliance with guidelines for animal care established by the Institute of Laboratory Animal Resources, National Research Council, U.S.A. (Institute of Laboratory Animal Research, 1996) and were approved by the Animal Care and Use committee at INIMEC-CONICET.

### Prenatal treatments

During GD 17–20 pregnant females received a daily intragastric administration of either 0 or 2 g/kg EtOH. Control dams (Prenatal Water) received 0.015 ml per g of body weight of tap water. The 2 g/kg EtOH dose was achieved using a similar volume (0.015 ml/g) of a 16.8% v/v EtOH solution (190 proof alcohol, Porta Hnos.). This EtOH dose and timing of exposure were chosen according to previous data showing that, at this age, the fetus can process EtOH chemosensory as well as its postabsorptive effects and form an associative memory comprising these stimuli (Abate et al., 2002; Chotro and Arias, 2003). During GD 21, pups were born by cesarean delivery procedure. For a more detailed description of procedures involved in prenatal treatment and cesarean delivery see (Domínguez et al., 1996; Abate et al., 2002). This procedure was performed to avoid suckling experiences with the dam. It has been shown that appetitive behavior toward the surrogate nipple increases over the first 3 h after birth (Smotherman et al., 1997) and that suckling from an artificial nipple is more vigorous when pups lack experience with the maternal nipple (Petrov et al., 2001). In Experiment 1, 12 pregnant females received water (group Prenatal Water) and 13 received EtOH (group Prenatal EtOH). For Experiment 2, prenatal pups from water treated dams were derived from 10 pregnant females and pups in the prenatal EtOH group were derived from 9 dams.

### Central drug administration procedure

Drugs were administered into the cisterna magna (intra-cisterna magna administration –IC-) using a 30-gauge hypodermic needle attached to transparent polyethylene tubing (PE 10, Clay Adams, Parsippany, NJ). EtOH and ACD were prepared using phosphate buffer as a vehicle (PB). For Experiment 1, vehicle (PB 0.1 M), EtOH (0.02 μmol) or ACD (0.35 μmol) were administered. The dosage of each particular drug was selected in accordance with previous literature (Arizzi-LaFrance et al., 2006; Nizhnikov et al., 2006c, 2007; Correa et al., 2009; March et al., 2013). For Experiment 2, the following ACD doses were administered: 0.0, 0.35 or 0.52 μmol. Administration procedure has been described elsewhere (Nizhnikov et al., 2006c; March et al., 2013). Briefly, the needle was inserted under visual guidance into the foramen magnum between the occipital bone and the first cervical vertebra (Petrov et al., 1998). Successful placement of the needle into the target site was confirmed by the appearance of cerebrospinal fluid in the tubing. The corresponding solution (1 μ l) was injected during a period of 10 s using a micrometer gastight syringe (Hewlett Packard, U.S.A.). Following each IC administration, the needle remained in position during 30 s and then removed to minimize leaking of cerebrospinal fluid. It has been observed that an inert substance administered into the cisterna magna (inulin) follows a caudal-to-rostral and ventral-to-dorsal pattern of distribution and preferred entry of tracer from ventral surfaces of the ventral forebrain—particularly hypothalamus—and brainstem (Proescholdt et al., 2000).

### Apparatus

In Experiment 1 and 2, neonates were kept in a heated incubator (32–34°C; Fábrica Eléctrica Delver, La Plata, Argentina) until commencement of experimental manipulations. Responsiveness to a surrogate nipple (Experiment 1) and motor reactivity (Experiment 2) were assessed 3–5 h after cesarean delivery. The evaluation procedure took place in a transparent Plexiglas (63 × 50 × 25 cm) glove box equipped with a fan system for ventilation and two holes in the front section that allowed access to the neonate. For facilitating presentation of the nipple or aromatic cues, newborns were individually placed in this conditioning chamber equipped with a heated Styrofoam container (internal base diameter: 9 cm; volume capacity: 750 cc) maintained at 35 ± 0.5°C via a temperature controller (Model 40-90B; Frederick Haer Co., Brunswick, ME).

### Assessment of ACD motivational effects by the artificial nipple technique (Experiment 1)

Two hours after delivery, pups received an intracisternal (IC) administration of one of the following drugs: vehicle (PB 0.1 M), EtOH (0.02 μmol), or ACD (0.35 μmol). Thirty seconds later (see drug administration procedure), pups were suited in a restriction vest and taken into the conditioning chamber. One minute after placement in the conditioning chamber, lemon odor (CS) was presented using a cotton applicator scented with 0.1 cc of lemon oil (Montreal, Argentina) during 5 min. Pups returned to the incubator where they remained for 1 h until commencement of the nipple attachment test.

During testing, pups were presented with an artificial nipple scented with lemon oil. Carved soft vinyl was used to shape a 25-mm long surrogate nipple also characterized by a rounded tip with a diameter of 1 mm. The base of the nipple was attached to the end of an angled dental probe, which served to allow precise control of the position of the nipple in relation to the pup and to establish physical distance between the newborn and the experimenter. Polyethylene tubing (PE 10, 0.58 mm inner diameter; Clay Adams, Sparks, MD) was inserted through the nipple. This tubing was attached to a syringe that contained distilled water. The tubing and the syringe, once filled with the distilled water, represented an open hydraulic system because the end of the tubing was opened and the syringe had a hole located in its upper body surface. Slight negative pressure produced by pups while attached to the nipple was necessary and sufficient to extract fluid from this device. Water availability through the nipple seems to facilitate attachment behavior but water does not induce conditioning in these circumstances (Smotherman et al., 1993). Before testing, pups were voided and body weights were registered. Immediately after termination of the test, body weights were also registered. An experimenter, blind to pre- and postnatal treatments, tested the pups via the artificial nipple technique. Exposure to the surrogate nipple involved gentle contact between the tip of the nipple and the oral area of the test subject. No attempt was made to force the tip of the nipple into the mouth of the pup (Petrov et al., 1997). Attachment was confirmed by periodic (every 30 s) gentle attempts to withdraw the nipple from the pup. The pup's active release of the nipple was considered to be a disengagement from the nipple (Nizhnikov et al., 2006c). Video records were obtained during conditioning and testing sessions.

### Assessment of motor reactivity following an IC administration of ACD in newborns (Experiment 2)

Two hours after delivery, pups prenatally exposed to 0 or 2 g/Kg EtOH, were IC administered with 0, 0.35 or 0.52 μmol ACD. One minute following ACD central administration, pups were placed in the conditioning chamber and were given five discrete presentations of lemon odor. Each odor exposure lasted for 5 s. One group of animals was presented with this odorant five times during the first 5 min of testing (1–5 min). These pups remained in the heated container for 5 additional minutes without further olfactory stimulation (Olfactory Treatment: Odor/No Odor). The remaining pups were placed in the heated container without lemon odor for the first 5 min, then were given five presentations of lemon odor distributed in 5 min (Olfactory Treatment: No Odor/Odor). Motor activity of all animals was videotaped.

### Experimental design and data analysis

Experiment 1 was a 2 (prenatal treatment: Water or EtOH) × 3 (US: vehicle, EtOH or ACD) between-subject factorial design. No more than one subject from a given litter was assigned to the same treatment condition (Holson and Pearce, 1992). Efforts were made to maintain an equivalent number of males and females per group. Number of pups per group was as follows: prenatal water/postnatal vehicle, *n* = 12; prenatal water/postnatal EtOH, *n* = 10; prenatal water/postnatal ACD, *n* = 8; prenatal EtOH/postnatal vehicle, *n* = 11; prenatal EtOH/postnatal EtOH, *n* = 10; and, prenatal EtOH/postnatal ACD, *n* = 8. During conditioning, duration and frequency of forelimb and hind limb movements were registered (results are shown in Table 2). During testing, the suckling response was further delineated separated into two components consisting of (1) measures of latency to grasp the nipple, total time spent on the nipple (referred as total attachment duration, calculated as the sum of the duration of all grasps), and mean grasp duration (total time divided by number of grasps), and (2) percentage of body weight gain (measure of fluid intake). Additionally, latency to perform limb movements during testing was also registered. All of these measures served as dependent variables.

Experiment 2 was defined by orthogonal variation in prenatal treatment (water or EtOH), postnatal ACD administration (0, 0.35, or 0.52 μmol), and order of odor presentation (groups No Odor/Odor and Odor/No Odor). Temporal block of testing (1–5 and 6–10 min) served as a within factor. Each group was composed by 7–9 pups. A total of 99 pups were utilized. Frequency of crawling, rolling, turning on side, probing and stretching were registered during the first 10 s of each minute of testing. Probing was registered when the neonate touched the rounded wall of the heated container with its nose. Stretching was considered as coordinated extension of both hindlimbs, often accompanied by dorsoflexion of the back and elevation of the head. Overall motor activity was considered as the sum of the frequency of the mentioned behaviors. Latency to show any of these behaviors was also registered (we will refer to this variable as latency to exert an overt behavior). Separated mixed ANOVAs were used to analyze motor activity and latency to exhibit an overt behavior.

Data were evaluated using separate between-groups ANOVA procedures. Significant interactions were further analyzed using Tukey's HSD tests with a probability of Type I error set at 0.05. In this and prior studies, it was observed that sex systematically failed to exert significant effects or to interact with EtOH reinforcement (Nizhnikov et al., 2012; Pautassi et al., 2012a,b,c). For this reason, inferential processing of data were performed by collapsing sex across treatments.

## Results

### Effects of prenatal EtOh exposure upon maternal and neonatal physical parameters

In Experiment 1, percentage of maternal body weight gained (%BWG) during gestational days 17–20, number of pups delivered alive and pup's body weight at birth were evaluated. Percentage of dam's %BWG during late pregnancy was calculated as follows: {[(maternal body weight at GD20—maternal body weight at GD17)/maternal body weight at GD17] × 100}. A One-Way ANOVA showed that prenatal treatments had no effect upon this index [*F*_(1, 23)_ = 0.10; *p* = 0.75]. Number of pups born alive was not affected by prenatal exposure to EtOH [*F*_(1, 23)_ = 0.93, *p* = 0.35]. On the other hand, pups' body weights at birth (averaged within each litter), were affected by prenatal treatment [*F*_(1, 23)_ = 5.95; *p* < 0.025]. Pups prenatally exposed to EtOH had lower body weights than control pups. These data have been summarized in Table 1.

**Table 1:** **Maternal and neonatal physical parameters registered in Experiments 1 and 2, as a function of prenatal treatments**.

	**Treatment during GD 17–20**			
	**Experiment 1**		**Experiment 2**	
	**Water**	**EtOH**	**Water**	**EtOH**
Percentage of MBW gain (g.)	7.78 ± 0.49	7.97 ± 0.36	8.22 ± 0.87	7.15 ± 0.92
Number of pups per litter	8.42 ± 0.79	9.46 ± 0.74	8.50 ± 0.91	9.77 ± 0.96
Litter's average weight (g.)	5.02 ± 0.11	4.74^*^ ± 0.04	5.05 ± 0.13	4.70 ± 0.14

Data are presented as mean ± SEM. Asterisk represent statistically different from water controls in the same experiment (p < 0.05).

In Experiment 2, neither percentage of maternal body weight gain during GDs 17–20, number of pups per litter, nor average litter body weight were significantly affected by prenatal treatment. Data have been summarized in Table 1.

### Assessment of ACD motivational effects by the artificial nipple technique (Experiment 1)

#### Conditioning session

Limb activity was registered during conditioning. Neither frequency [*F*_(2, 53)_ = 0.84, *p* = 0.44] of limb movement nor duration [*F*_(2, 53)_ = 0.13, *p* = 0.87] differed across treatments. These data have been summarized in Table 2.

**Table 2:** **Data summarize mean + SEM duration and frequency of limb movements registered during the conditioning phase in Experiment 1**.

**US drug**	**Prenatal treatment**			
	**Water**		**EtOH**	
	**Frequency**	**Duration**	**Frequency**	**Duration**
Vehicle	12.08 ± 2.06	144.85 ± 27.09	9.90 ± 2.15	95.06 ± 28.30
EtOH	11.90 ± 2.26	140.47 ± 29.68	12.50 ± 2.26	118.00 ± 29.68
ACD	14.25 ± 2.53	135.16 ± 33.18	8.62 ± 2.53	86.92 ± 33.18

#### Attachment behavior

**Figure 1:**
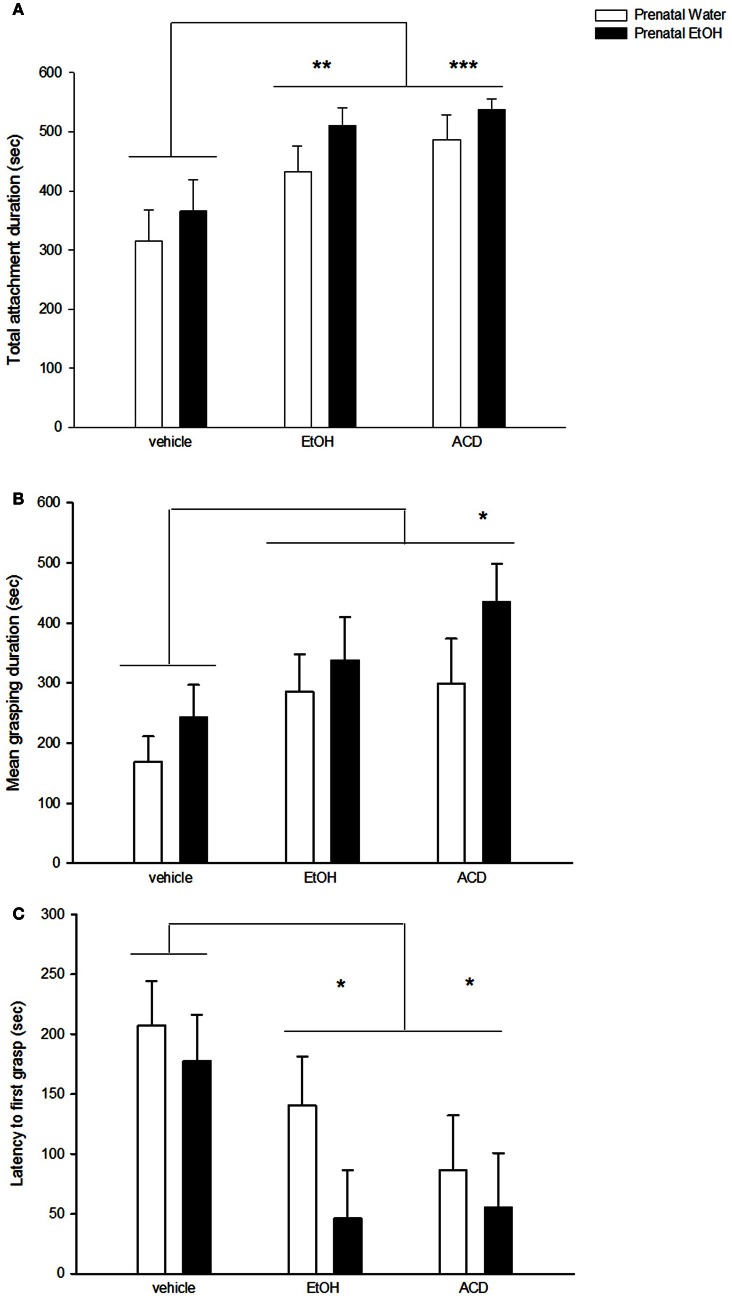
**Effect of prenatal exposure to EtOH on attachment behavior to a surrogate nipple in the presence of an odor conditioned to EtOH or ACD**. Data represent mean + SEM in seconds of **(A)** total attachment duration, **(B)** grasp duration, and **(C)** latency to first grasp. Asterisk (^*^p < 0.05; ^**^p < 0.01; and ^***^p < 0.001) depicts a significant difference from vehicle control group.

Conditioned reinforcing effects of EtOH and ACD were observed in the analysis of total attachment duration [main effect of US drug: *F*_(2, 53)_ = 8.27; *p* < 0.001]. This dependent variable was significantly higher in neonates conditioned with EtOH (*p* < 0.01) or ACD (*p* < 0.001) compared to control pups. A similar profile was found when analyzing mean grasping duration [main effect of US drug: *F*_(2, 53)_ = 3.65; *p* < 0.05]. In this case, EtOH-treated pups displayed intermediate levels of mean grasping duration, whereas those pups treated with ACD differed significantly from the control group (*p* < 0.05). Additionally, latency to grasp the nipple varied as a function of drug utilized as US [*F*_(2, 53)_ = 5.16, *p* < 0.01]. Neonates from the control group had longer delays to grasp the artificial nipple than did EtOH (*p* < 0.05) or ACD (*p* < 0.025) treated pups. These results have been summarized in Figure 1.

Latency to initiate hindlimb or forelimb movements were recorded during testing. Latency to initiate limb movement was not affected by prenatal treatment or by the drug given during conditioning [*F*_(2, 53)_ = 0.65, *p* = 0.53]. Means and S.E. of the mean were as follow: for neonates prenatally treated with water and postnatally administered with vehicle: 26.73 ± 4.86, EtOH: 15.51 ± 5.52 and ACD: 25.01 ± 5.95 and for neonates prenatally exposed to EtOH and postnatally administered with vehicle: 19.27 ± 5.07, EtOH: 16.45 ± 5.32, and ACD: 13.67 ± 5.95.

#### Intake

%BWG varied as a function of drug administered during conditioning [main effect: *F*_(2, 53)_ = 6.45, *p* < 0.01]. *Post-hoc* testing indicated that neonates conditioned with EtOH, achieved higher %BWG than control siblings (*p* < 0.001). Neonates conditioned with ACD had intermediate levels of water consumption (means and S.E. were as follows: 0.17 ± 0.07 g; 0.51 ± 0.07 g and 0.38 ± 0.08 g for the control, EtOH and ACD groups, respectively). Prenatal treatment did not exert any significant effect upon this dependent variable nor did it interact with postnatal treatment.

### Motor reactivity following central administration of ACD as a function of prenatal treatment (Experiment 2)

**Figure 2:**
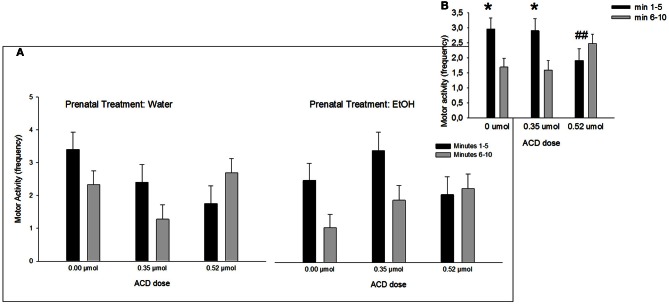
**Effect of central ACD administration on motor activity**. Data represent mean + SEM of motor behavior (frequency) in consecutive 5 min blocks during testing. **(A)** Motor activity after central ACD administration as a function of prenatal exposure to EtOH. **(B)** Motor activity collapsed across prenatal treatments. Asterisk (^*^p < 0.01) depicts a significant difference between time intervals in the same ACD dose. Double numeral (^##^*p* < 0.01) indicates a significant difference between doses during minutes in block 1–5 min.

A Four-Way ANOVA (prenatal treatment × ACD dose × olfactory treatment × time block) showed that frequency of motor activity was significantly higher during the first time block of testing relative to the second block [*F*_(1, 86)_ = 10.21, *p* < 0.01]. Interestingly, this effect was tempered by the interaction involving block and ACD treatment [*F*_(2, 86)_ = 8.58, p < 0.001]. This results have been depicted in Figure 2A. *Post-hoc* analysis indicated that while levels of activity during the first, relative to the second block of testing, were significantly higher in pups administered 0 or 0.35 μmol ACD (both *p*'s < 0.01). This difference was not significant in neonates that received the highest ACD dose (0.52 μmol). In this group, levels of activity remained low across testing. This significant interaction is depicted in Figure 2B. When analyzing this dependent variable, prenatal treatment did not exert statistically significant effects.

**Figure 3:**
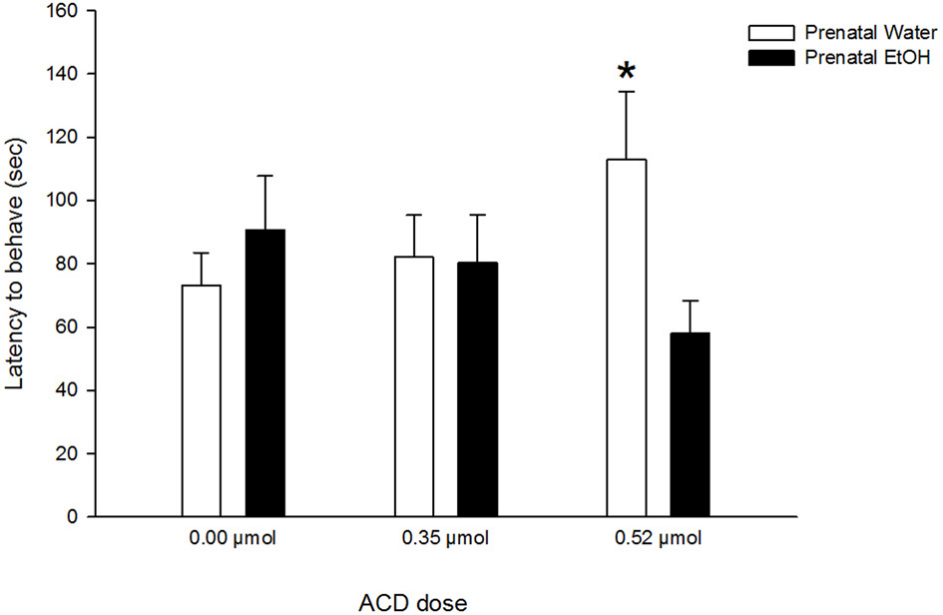
**Effect of prenatal exposure to EtOH on latency to exert a given overt behavior after central ACD administration**. Data represent mean + SEM in seconds. Asterisk (^*^p < 0.025) depicts significant differences between prenatal treatments in the same ACD dose.

A significant main effect of olfactory treatment was found in the analysis of latency to exert an overt behavior. Specifically, pups exposed to lemon odor at commencement of testing (Odor/No Odor group: 70 ± 8 s) exhibited significantly lower latency scores than pups initially exposed to a non-olfactory testing context (No Odor/Odor group: 96 ± 8 s). Presentation of lemon odorant rapidly recruited behavioral responsiveness. A significant interaction between prenatal and ACD treatments [*F*_(2, 86)_ = 3.19, *p* < 0.05] was found. *Post-hoc* comparisons indicated that neonates treated with the highest ACD dose (0.52 μmol) and with no prior EtOH experience exhibited significantly longer latencies (*p* < 0.025) than pups treated with a similar ACD dose but with a positive history of prenatal EtOH exposure (Figure 3).

## Discussion

As mentioned, EtOH exposure during gestation has been linked to later EtOH acceptance and drinking. In previous studies, we have found that EtOH exposure during GD 17–20 increases postnatal operant responsiveness for an intraorally infused EtOH solution (3% v/v) (March et al., 2009; Miranda-Morales et al., 2010). Nizhnikov et al. (2006a) found that prenatal exposure to the drug also increased the range of doses capable of sustaining appetitive conditioning when EtOH is administered into the peritoneum. This route of administration minimizes the possibility of recruiting the chemosensory properties of the drug. Nevertheless, some detection of such cues can be present due to hematopoietic stimulation (Nizhnikov et al., 2006a). In Experiment 1, when EtOH and ACD were directly administered in the cisterna magna, differences in drug induced appetitive conditioning as a function of prenatal exposure to EtOH were not observed. However, only one dose of each substance was tested in the mentioned experiment. It is possible that an effect of prenatal exposure to EtOH upon central reinforcement of ACD might emerge when using a wider range of doses.

It could be objected that in the present study we did not employ untreated dams during gestation, since prenatal stress by itself can alter later responsiveness to EtOH (Van Waes et al., 2011a,b). However, in a recent study (March et al., 2013) we analyzed central EtOH and ACD appetitive conditioning in neonates using the artificial nipple technique. This study was conducted without any prior prenatal treatment and the results in terms of conditioned responses, promoted by the different pharmacological treatments, is analogous to those reported in the present article.

It can be argued that differences in suckling from the artificial nipple in neonates administered with EtOH or ACD compared to vehicle-administered siblings, can be explained either by pseudo-conditioning or by the effects of these substances upon motor activity. Regarding the first possibility, previous studies in which unpaired and US-only control groups have been included have consistently supported the notion that an associative learning mechanism underlies later increases in responses to lemon-nipple-water CS when EtOH is used as the US (Petrov et al., 2003; Nizhnikov et al., 2006b,c). The second alternative explanation can arise from the observation that in preweanlings, motor conditioned responses arising from the use of EtOH as a US can confound interpretations in tests related with drug reinforcement (Molina et al., 2007a). In Experiment 1 we directly assessed limb activity during conditioning and found no differences between groups. Additionally, in Experiment 2 we explicitly examined in freely moving neonates if ACD had specific motor effects in a time frame similar to the post-administration time during which conditioning occurred in Experiment 1. Groups administered with 0.35 μmol of ACD did not show any modifications in motor activity (whereas a higher dose did alter motor activity). This observation argues against the possibility that the behavioral expression of EtOH- or ACD-derived associative learning was due to motor effects of these pharmacological agents rather than their motivational properties.

Experiment 2 suggested that 0.52 μmol ACD had a sedative effect expressed as increased latency to display an overt behavior as well as a reduction in motor activity. These effects were not present in newborns prenatally exposed to EtOH, perhaps due to the development of tolerance to ACD effects. In adult rats and mice, increased locomotion has been induced by acute challenges with central ACD (in rats; Correa et al., 2003a,b, 2009; Sanchez-Catalan et al., 2009); in mice: Correa et al., 1999, 2001a. Sedative effects of ACD have been found in adult mice (Holtzman and Schneider, 1974; Correa et al., 2001b; Quertemont et al., 2004; Tambour et al., 2006, 2007) and, to a minor extent, in adult rats (Myers et al., 1987). In infant rats, ACD seems to stimulate motor activity (Pautassi et al., 2011). Current discrepancies concerning stimulatory vs. sedative effects in adults are difficult to reconcile due to differences in methodologies for inducing ACD, including direct administration of ACD vs. alteration of EtOH metabolism, as well as differences in route of administration and strain of rodent.

In summary, the present study found that very early in ontogeny (1) classical olfactory conditioning occurs when either EtOH or ACD are used as US, (2) this conditioning is expressed as an increase in suckling and in water ingestion from an artificial nipple scented with the odorant previously paired with intracisternal ACD (0.35 μmol) or EtOH and (3) motor activity, when the animals are allowed to move freely in the conditioning context, is decreased after an acute challenge with a high dose of ACD (0.52 μmol). This later effect emerged in animals without a prenatal history of EtOH exposure, whereas for animals prenatally exposed to EtOH, no sedative effect upon motor activity was observed. It is important to note that the emergence of sedative effects appears to coincide with the perception of aversive interoceptive effects of the drug while resistance to this effects has been linked to higher susceptibility to EtOH reinforcement (Arias et al., 2009). If prenatal EtOH exposure reduces sedative effects of the drug it may also ameliorate its aversive properties. In turn, this might help explain the heightened disposition to consume EtOH observed in prenatally exposed subjects and their sensitivity to alternative positive effects of the drug.

There is some controversy in the literature regarding the effect of central ACD in mediating EtOH effects. Uncertainty originates from the observation that very low ACD is detected in the brain after EtOH administration (Gill et al., 1992; Hunt, 1996). However, the participation of ACD has been shown not only when directly administering this substance in the brain (Rodd-Henricks et al., 2002; Correa et al., 2003b, 2009) but also by manipulating EtOH metabolism (Arizzi-LaFrance et al., 2006; Font et al., 2006; Correa et al., 2008; Pastor and Aragon, 2008; Enrico et al., 2009). In the present study, from a behavioral perspective, EtOH and ACD exerted a similar magnitude of appetitive conditioning. Also, we have previously observed that EtOH and ACD reinforcement is similarly inhibited by sequestering ACD trough d-penicillamine (March et al., 2013).

The role of ACD and acetate in mediating EtOH postabsortive effects has been widely studied in adult animals. However, we should be cautious in extrapolating findings in adult animals to expected results in newborns since there are marked differences in metabolic systems (both peripheral and central) between these ontogenetic stages. Catalase concentrations in cerebellum, striatum, cerebral hemispheres, and brain stem of the newborn rat are about eight times higher than those observed in the adult organism (Del Maestro and McDonald, 1987). Additionally, pre-weanlings have slower rates of EtOH blood metabolism after systemic administration compared to older animals (Silveri and Spear, 2000). In newborn and infantile rats, ACD-mediated positive reinforcement and behavioral activation has been observed (Nizhnikov et al., 2007; Pautassi et al., 2011). Nevertheless, the profile of behavioral effects derived from ACD has not been extensively studied during early ontogeny. For example, negative reinforcement, a property that is believed to play an important role in EtOH use and abuse, has yet to be directly assessed. The gap in contemporary knowledge of the role of ACD in EtOH's postabsortive effects at this developmental period emphasizes the importance of the present study as well as the need for further tests of ontogenetic differences in EtOH acceptance and the role of its metabolites in these differences.

### Conflict of interest statement

The authors declare that the research was conducted in the absence of any commercial or financial relationships that could be construed as a potential conflict of interest.
